# Change in Serum Uric Acid is a Useful Predictor of All-Cause Mortality among Community-Dwelling Persons

**DOI:** 10.1155/2023/7382320

**Published:** 2023-03-04

**Authors:** Ryuichi Kawamoto, Asuka Kikuchi, Daisuke Ninomiya, Teru Kumagi

**Affiliations:** ^1^Department of Community Medicine, Ehime University Graduate School of Medicine, Ehime, Japan; ^2^Department of Internal Medicine, Seiyo Municipal Nomura Hospital, Seiyo, Japan

## Abstract

There is limited research on the association between longitudinal variability in serum uric acid (SUA) and all-cause mortality in the general population, although recent studies have suggested that changes in SUA are associated with all-cause mortality in adults. This study aims to examine the association between percentage change in SUA (%dSUA = 100 × (cohort 2 SUA − cohort 1 SUA)/(time × cohort 1 SUA) and all-cause mortality. This study is based on 1,301 participants, of whom 543 were male (63 ± 11 years) and 758 were female (63 ± 9 years). We obtained adjusted relative risk estimates for all-cause mortality and used a Cox proportional hazards model, adjusted for possible confounders, to determine the hazard ratio (HR) and 95% confidence interval (CI) of %dSUA. Of all the participants, 79 (6.1%) were deceased, and of these, 45 were male (8.3%) and 34 were female (4.5%). The multivariable-adjusted HRs (95% CI) for all-cause mortality for the first, second to fourth (reference), and fifth %dSUA quintiles were 3.79 (1.67–8.48), 1.00, and 0.87 (0.29–2.61) for male participants and 4.00 (1.43–11.2), 1.00, and 1.19 (0.46–3.05) for female participants, respectively. Participants with a body mass index of <22 kg/m^2^ had a significantly higher HR, forming a *U*-shaped curve for the first (HR, 7.59; 95% CI, 2.13–27.0) and fifth quintiles (HR, 2.93; 95% CI, 1.05–8.18) relative to the reference. Percentage change in SUA is independently and significantly associated with future all-cause mortality among community-dwelling persons.

## 1. Introduction

Uric acid is the final oxidation product of purine metabolism in humans. Xanthine oxidase is critical for producing uric acid since it breaks down purine nucleotides. According to experimental and epidemiologic research, increased levels of serum uric acid (SUA) are associated with hypertension [1–3], metabolic syndrome [4, 5], and cardiovascular disease (CVD) incidence [6, 7]. Furthermore, it detrimentally affects the longevity of individuals with these conditions. Some studies have indicated that the relationship between SUA levels and mortality is *U*-shaped for both genders [8, 9], particularly females [10], whereas others have suggested a *U*-shaped relationship for both genders [11–13], specifically males [10]. These inconsistent results can be attributed to differences in factors such as gender, age, race, body mass index (BMI), medication, renal function, underlying diseases, and stage of the disease. Furthermore, most studies are based on a single baseline SUA measurement and thus may not reflect the association between mortality and risk of chronic SUA exposure [14].

More recent clinical observations have shown that increased variability in BMI [15], blood glucose (BG), and SUA [16–20] are associated with an increased risk of all-cause mortality. Variations in hemodynamic and metabolic parameters have been associated with poor prognosis. Tian et al. [20], for example, show an independent association between variability in SUA levels and greater risk of all-cause mortality, irrespective of baseline SUA and the direction of variability in the general population. However, it is not known whether this presumed relationship varies with the change in SUA levels.

Therefore, the purpose of our study was to examine the relationship between changes in SUA levels and potential risk factors such as gender, BMI, hypertension, hyperglycemia, lipids, renal dysfunction, and all-cause mortality using cohort data for community-dwelling persons.

## 2. Materials and Methods

### 2.1. Study Design and Participants

This research is a prospective cohort analysis that is based on data from the Nomura study, conducted in 2002 (cohort 1) and 2014 (cohort 2) [21]. The participants were rural residents of Seiyo City who underwent a community-based annual health examination. A flowchart of participant enrollment and exclusion is presented in a previous study [21]. In brief, 3,553 participants aged 19–90 years, of whom 1,573 were male and 1,980 were female, enrolled for a community-based health examination. The data collected included demographic and clinical indicators such as age, gender, smoking habits, alcohol consumption, CVD history, medical history, and the results of clinical examinations and laboratory tests. Followup studies were conducted after 19 years for the first group and seven years for the second. The participants' survival status was obtained from the Japanese Basic Resident Registry. For this study, participants in both cohorts who underwent followup examinations for changes in SUA levels were included. The first cohort included 651 such participants and the second included 650. Data for both cohorts (*N* = 1,301) were analyzed. All participants were in the age range of 24–88 years when they enrolled in the study. The study was reviewed and approved by the institutional review board of Ehime University Hospital (1903018). All participants provided written informed consent.

### 2.2. Measurement of Percentage Change in SUA

Measurements of SUA levels obtained during the first visit are denoted SUA1, and those obtained during the second visit are denoted SUA2. The percentage difference between the two values is represented by %dSUA, which is estimated as 100 × (SUA2 − SUA1)/{time (in years) × SUA1}.

### 2.3. Evaluation of Risk Factors

The baseline anthropometric indices measured were weight and height. The participants' BMI was calculated as weight (kg) divided by height squared (m^2^). Smoking status (pack-years) was the product of the number of years a participant had been a smoker and the average number of packs per day. Based on this measure, the participants were categorized as nonsmokers, ex-smokers, light smokers (<20 pack-years), or heavy smokers (>20 pack-years). Similarly, daily alcohol intake was estimated based on the Japanese liquor unit (22.9 g ethanol, equivalent to a bottle of sake). Participants were classified as nondrinkers, occasional drinkers (<1 unit/day), daily light drinkers (1-2 units/day), or daily heavy drinkers (2-3 units/day). No participants drank more than 3 units/day. Systolic blood pressure (SBP) and diastolic blood pressure (DBP) were measured twice for each participant using an automatic oscillometric blood pressure recorder (BP-103i; Colin, Aichi, Japan). Prior to the measurement, participants were requested to rest for at least five minutes and remain in a seated position. We used the means of two measurements in our analysis. The participants were also asked to fast overnight, so that blood samples collected could be examined for triglycerides (TG), high-density lipoprotein cholesterol (HDL-C), low-density lipoprotein cholesterol (LDL-C), SUA, BG, and creatinine (Cr). The estimated glomerular filtration rate (eGFR) was calculated using the chronic kidney disease epidemiology collaboration (CKDepi) equation (mL/min/1.732 m^2^) with the following coefficients, calculated for the Japanese population. Males: Cr ≤ 0.9 mg/dL, 141 × (Cr/0.9)^−0.411^ × 0.993^age^ × 0.813; Cr > 0.9 mg/dL, 141 × (Cr/0.9)^−1.209^ × 0.993^age^ × 0.813; females: Cr ≤ 0.7 mg/dL, 144 × (Cr/0.7)^−0.329^ × 0.993^age^ × 0.813; Cr > 0.7 mg/dL, 144 × (Cr/0.7)^−1.209^ × 0.993^age^ × 0.813 [22].

Participants with SBP ≥140 mmHg and DBP ≥90 mmHg or who were taking antihypertensive medication were classified as having hypertension. Participants with TG levels ≥150 mg/dL were classified as having hypertriglyceridemia; those with HDL-C levels <40 mg/dL were considered to have low HDL cholesterol; and those with LDL-C levels ≥140 mg/dL or on antidyslipidemic medication were categorized as having hyper LDL-cholesterolemia. Participants with BG ≥ 126 mg/dL or on antidiabetic medication were classified as diabetic. Males with SUA ≥6.0 mg/dL and females with SUA ≥5.0 mg/dL were classified as having hyperuricemia [9]. An eGFR of <60 mL/min/1.73 m^2^ was considered an indicator of chronic kidney disease (CKD). Ischemic heart disease, ischemic stroke, and peripheral vascular disease were classified as CVD.

### 2.4. Statistical Analysis

We conducted the statistical analysis using IBM SPSS Statistics (version 26.0; SPSS, Chicago, IL, USA). Normally distributed continuous variables were expressed as mean ± standard deviation (SD), and non-normally distributed variables (e.g., TG and BG) were expressed as median and quartiles. Log-transformed values were used for parameters with nonnormal distributions. The participants were divided into three groups according to %dSUA quintiles (quintile 1: <−1.88%/year; quintile 2–4: −1.87 to 2.50%/year; quintile 5: ≥2.51%/year). Categorical variables were compared by conducting a chi-square analysis, and continuous variables were compared by performing a student's *t*-test on normally distributed variables. A Cox proportional hazards regression was to investigate the factors associated with all-cause mortality and model the relationships between %dSUA and all-cause mortality. We used age as the time axis and adjusted for baseline characteristics and confounding factors such as age, BMI, smoking and drinking habits, history of CVD, hypertension, hypertriglyceridemia, low HDL cholesterol, hyper-LDL cholesterolemia, diabetes, and CKD. Consistency in the observed association between %dSUA and all-cause mortality was determined by performing subgroup analyses. All significant confounding variables except the effect and effect variables were adjusted for in the interaction tests. All *p* values were two-tailed, and *p* < 0.05 was considered significant.

## 3. Results

### 3.1. Participants' Baseline Characteristics According to %dSUA Quintiles

Male participants accounted for 41.7% (543) of the total sample (1,301 participants). The mean (±SD) age for male participants was 63 (±11) years, and that for female participants was 63 (±9) years. As shown in Table 1, participants in the first %dSUA quintile were older; were more likely to be smokers; were less likely to be drinkers; had a higher prevalence of hypertension, hyper LDL-cholesterolemia, CKD, and hyperuricemia and were more likely to be on antihypertensive and antilipidemic medication. The results also revealed that higher SUA levels were associated with lower %dSUA quintiles. There was no significant association, however, between %dSUA quintiles and the prevalence of CVD, hypertriglyceridemia, lower HDL-cholesterolemia, or diabetes.

### 3.2. Kaplan–Meier Curve for All-Cause Mortality According to %dSUA Quintiles by Gender

**Figure 1:**
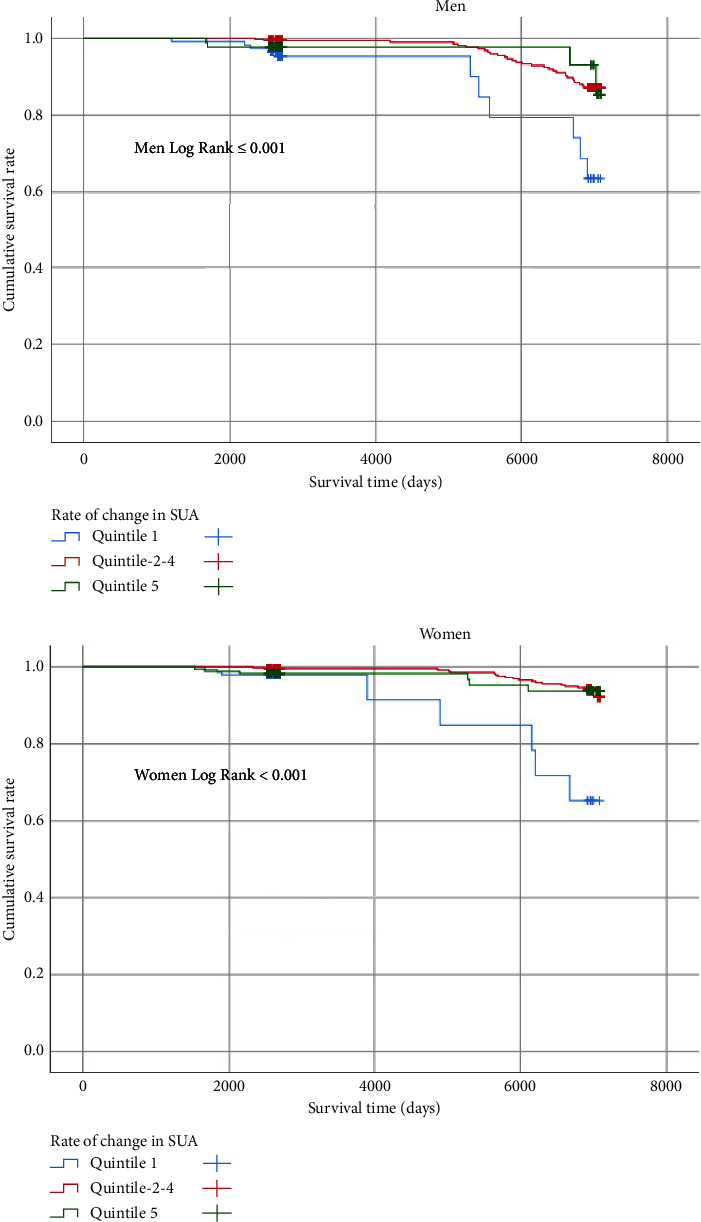
Kaplan–Meier curves for all-cause mortality among men and women by quintiles of percentage change in SUA.

A total of 79 subjects were reported to have died during the median followup period of 10.7 years (interquartile range: 7.3–19.1). The incidence rate for all-cause mortality decreased from 8.46 per 1,000 person-years for the lowest quintile to 4.20 for the second to fourth quintiles and 3.45 for the highest quintile. In Figure 1, Kaplan–Meier estimates suggested that individuals of both genders in the first %dSUA quintile were at a higher risk of all-cause mortality than other participants during the 10.7-year followup period (log-rank test, *p* ≤ 0.001).

### 3.3. Hazard Ratios for All-Cause Mortality According to %dSUA Quintiles by Gender and BMI

As shown in Table 2, for both genders, the model indicated that participants in the first %dSUA quintile were at a significantly higher risk of all-cause mortality than those in the second to fourth %dSUA quintiles, which were used as the reference. The multivariable-adjusted HRs (95% CI) for all-cause mortality across the first, second to fourth, and fifth %dSUA quintiles were 3.79 (1.67–8.48), 1.00, and 0.87 (0.29–2.61) for male participants, and 4.00 (1.43–11.2), 1.00, and 1.19 (0.46–3.05) for female participants.

### 3.4. Hazard Ratios for All-Cause Mortality According to %dSUA Quintiles by Subanalysis

In Table 3, participants were stratified by age (<65 and ≥65 years), BMI (<22.0 and ≥22.0 kg/m^2^), medication (absence and presence of antihypertensive, antidyslipidemic, or antidiabetic medication), eGFR (<60 and ≥60 mL/min/1.73 m^2^/y), and SUA (<6.0 and ≥6.0 mg/dL for males and <5.0 and ≥5.0 mg/dL for females). Overall, the results showed that the first %dSUA quintile was significantly associated with a higher risk of all-cause mortality. Participants with a BMI of <22.0 kg/m^2^ exhibited significantly higher HRs, forming a *U*-shaped curve for the first (HR, 7.59; 95% CI, 2.13–27.0) and the quintiles (HR, 2.93; 95% CI, 1.05–8.18), compared with the reference (the second to fourth quintiles). For participants with BMI ≥22.0 kg/m^2^, the multivariable-adjusted HRs (95% CI) for all-cause mortality across the first, second to fourth, and fifth quintiles of %dSUA were 3.08 (1.46–6.48), 1.00, and 0.42 (0.13–1.42), respectively.

## 4. Discussion

This prospective followup study was designed to examine the relationship between potential confounders, including percentage change in SUA levels and all-cause mortality, using data for 1,301 community dwellers. The SUA levels were measured twice for each participant, and data for all-cause mortality were obtained from Japan's Basic Resident Registry. The results indicated the existence of a significant and independent association between a decrease in SUA and all-cause mortality. In participants with a BMI of <22.0 kg/m^2^, we observed a *U*-shaped relationship, in that a positive %dSUA was associated with a significantly higher HR for all-cause mortality. To the best of our knowledge, few studies have indicated percentage change in SUA as an important risk factor for all-cause mortality among community-dwelling persons [20].

There are not many reports of SUA fluctuations playing an important role in increasing disease risk [18–20]. Reduced SUA levels in patients with gout may be associated with a lower risk of renal function decline but not with a lower risk for diabetes or CVD [23]. A study conducted on 3,604 male participants aged 45–74 years who enrolled in one of the three MONICA Augsburg surveys during 1984–1995 reported 809 total deaths [24]. A Cox model comparing the extreme quartiles of SUA distribution and all-cause mortality reported an HR of 1.40 (95% CI, 1.13–1.74) after adjusting for conventional CVD risk factors and diuretic intake. Tian et al. [14] examined 63,127 participants without a history of CVD and showed that changes in SUA at either extreme were associated with a higher risk of all-cause mortality. Their study showed HRs (95% CIs) of 1.15 (1.02–1.29) and 1.20 (1.06–1.35) for the first and fifth quintiles. Our research shows that a decrease or increase in SUA of more than 20% is associated with an increased risk of all-cause mortality. In 309 peritoneal dialysis patients who were not on SUA-lowering medication, there was a higher mortality rate among those whose SUA levels dropped (19 out of 86) than among those whose SUA levels were nondecliner (3 out of 86; *p* < 0.001). Furthermore, a Cox regression analysis revealed SUA decline as an independent risk factor for all-cause mortality [16]. Savarese et al. [25] conducted a meta-regression analysis on data for 21,373 participants who were part of 11 trials with a mean follow-up period of 2.02 ± 1.76 years, which included 4,533 cardiovascular events. This analysis revealed no relationship between change in SUA from baseline to the end of the followup period and the study's composite outcome or all-cause mortality [25]. It is therefore yet to be convincingly established whether long-term fluctuations in SUA are associated with the risk of all-cause mortality in the general population.

In addition, compared with participants who had a stable SUA, those whose SUA dropped dramatically were older; had a higher BMI and BG; had a higher incidence of hypertension, hyper LDL-cholesterolemia, and hyperuricemia; and had lower eGFR values. These parameters are indicative of cardiometabolic disorders, systemic inflammation, and poor renal function and may have an important influence on the pathogenesis of mortality [15, 17, 26, 27]. The relationship between %dSUA and these parameters may suggest a pathway via which SUA variability affects the risk of all-cause mortality.

Research is yet to provide a comprehensive understanding of the mechanisms underpinning increased all-cause mortality in individuals with fluctuating SUA levels. Uric acid is catalyzed by the enzyme xanthine oxidase, which is harmful to free radicals and has dual pro-oxidant and antioxidant properties [24]. Thus, excessive oxidative stress due to varying SUA levels may lead to induced endothelial dysfunction, affect the extent of activation of the renin-angiotensin system and indirectly contribute to the increased risk of all-cause mortality [28, 29]. In addition, it is known that a rapid increase in SUA increases the percentage of urate crystallization and promotes immune and inflammatory responses [30]. Moreover, studies have shown that SUA values are positively correlated with albumin and negatively correlated with the Charlson Comorbidity Index [31]. Thus, the increased risk of mortality associated with low SUA values could be attributable to a poor nutritional status associated with hypoalbuminemia, and the more severe this comorbidity is, the higher the risk of all-cause mortality. In our study, we observed a significantly higher HR for participants with a BMI of <22.0 kg/m^2^, forming a *U*-shaped curve. That was the greater the percentage increase and decrease in SUA, which showed fluctuating SUA levels, the higher the mortality rate.

Further research is needed to identify a clear mechanism to explain this. A key contribution of this research is the prospective design that can be attributed to the long-term study period, which included the followup analyses. Other advantages include measurements of SUA variability, adjustment for several possible confounding factors, and the inclusion of sensitivity analyses. However, our study is also subject to several limitations. First, the sample consisted primarily of relatively healthy middle-aged and older adults (mean age 68 ± 10 years) who lived in rural areas of Japan, where the population is rapidly aging, and who participated in health examinations. In addition, the subjects were cohort participants, and two medical examinations were needed to obtain data on their SUA variability. As a result, only a third of the cohort subjects were included in our analysis, so selection bias cannot be ruled out. Second, we used the all-cause mortality rate as the outcome, based on Japan's Basic Resident Register. However, this register does not contain data on participants who left the region during the study period, limiting the possibility of followups. Third, future research should consider the impact of changes in confounding factors, medication, underlying diseases, and lifestyle, both at baseline assessments and during followup periods. Fourth, we assessed renal function based only on eGFR and not using data on urinary albumin or protein. Finally, the relatively small number of participants and deaths may have weakened the causal relationship between %dSUA and all-cause mortality.

## 5. Conclusion

This study demonstrated that a decreased percentage change in SUA is strongly associated with all-cause mortality irrespective of gender and baseline covariates among community-dwelling persons. In addition, it revealed a relationship between increased percentage change in SUA and a significantly higher HR of all-cause mortality in participants with a BMI of <22 kg/m^2^. These findings highlight the importance of achieving stable SUA levels and avoiding large fluctuations in SUA levels and may inform the design of future studies to identify and treat true high-risk populations. Further research is needed to evaluate the reproducibility of our results and to further elucidate associations among the tested conditions.

## Figures and Tables

**Table 1:** Baseline characteristics according to %dSUA quintiles.

Baseline characteristics *N* = 1,301	%dSUA quintiles			*P* value
	Quintile 1	Quintiles 2–4	Quintile 5	
	<−1.88% (year)	−1.87 to 2.50% (year)	≥2.51% (year)	
	*N* = 261	*N* = 779	*N* = 261	
Men, *N* (%)	114 (43.7)	343 (44.0)	86 (33.0)	**0.006**
Age (years)	67 ± 9	61 ± 9	65 ± 10	**<0.001**
Body mass index (kg/m^2^)	23.1 ± 2.7	23.1 ± 3.1	22.6 ± 2.8	**0.026**
Smoking status^†^ (%)	51.3/20.7/10.7/17.2	68.8/14.4/5.6/11.2	52.8/17.2/8.0/11.9	**<0.001**
Drinking status^‡^ (%)	65.9/23.0/4.6/6.5	46.5/28.5/14.2/10.8	68.2/21.1/5.4/5.4	**<0.001**
Cardiovascular disease, *N* (%)	18 (6.9)	30 (3.9)	14 (5.4)	0.119
Hypertension, *N* (%)	171 (65.5)	348 (44.7)	149 (57.1)	**<0.001**
Systolic blood pressure (mmHg)	135 ± 18	132 ± 19	136 ± 20	**0.012**
Diastolic blood pressure (mmHg)	79 ± 10	79 ± 11	78 ± 11	0.147
Antihypertensive medication, *N* (%)	113 (43.3)	163 (20.9)	97 (32.7)	**<0.001**
Hypertriglyceridemia, *N* (%)	49 (18.8)	126 (16.2)	31 (11.9)	0.090
Triglycerides (mg/dL)	91 (67–130)	91 (68–124)	84 (63–114)	0.128
Low HDL cholesterolemia, *N* (%)	5 (1.9)	30 (3.9)	6 (2.3)	0.204
HDL cholesterol (mg/dL)	65 ± 15	64 ± 16	65 ± 16	0.445
Hyper LDL cholesterolemia, *N* (%)	124 (47.5)	261 (33.5)	102 (39.1)	**<0.001**
LDL cholesterol (mg/dL)	123 ± 32	120 ± 30	120 ± 30	0.378
Antidyslipidemic medication, *N* (%)	61 (23.4)	73 (9.4)	40 (15.3)	**<0.001**
Diabetes, *N* (%)	16 (6.1)	54 (6.9)	25 (9.6)	0.261
Blood glucose (mg/dL)	117 (108–126)	100 (90–114)	114 (99–121)	**<0.001**
Antidiabetic medication, *N* (%)	19 (7.3)	47 (6.0)	27 (10.3)	0.064
Chronic kidney disease, *N* (%)	31 (11.9)	46 (5.9)	18 (6.9)	**0.006**
eGFR (mL/min/1.73 m^2^)	72.6 ± 12.1	80.9 ± 15.1	77.4 ± 14.3	**<0.001**
Hyperuricemia, *N* (%)	138 (52.9)	304 (39.0)	53 (20.3)	**<0.001**
SUA (mg/dL)	5.5 ± 1.4	5.1 ± 1.3	4.5 ± 1.2	**<0.001**

%dSUA, percentage change in serum uric acid; eGFR, estimated glomerular filtration rate; HDL, high-density lipoprotein; LDL, low-density lipoprotein. ^†^Smoking status was defined as the number of cigarette packs per day multiplied by the number of years smoked (pack-year), and the participants were classified as nonsmokers, ex-smokers, light smokers (<20 pack-years), or heavy smokers (≥20 pack-years). ^‡^Alcohol consumption was measured using the Japanese liquor unit, in which a unit corresponds to 22.9 g of ethanol, and the participants were classified as nondrinkers, occasional drinkers (<1 unit/day), daily light drinkers (1-2 units/day), or daily heavy drinkers (2-3 units/day). Data presented are means ± standard deviation. Data for triglycerides and blood glucose were skewed, are presented as median (interquartile range) values, and were log-transformed for analysis. *P* values: ANOVA for continuous variables or the *χ*^2^-test for categorical variables. Bolded numbers indicate significance (*p* < 0.05).

**Table 2:** Hazard ratios and 95% CIs for all-cause mortality according to %dSUA quintiles stratified by gender.

Baseline characteristics	%dSUA quintiles			*P* for trend
	Hazard ratio (95% CI)			
	Quintile 1	Quintiles 2–4	Quintile 5	
	<−1.88% (year)	−1.87 to 2.50% (year)	≥2.51% (year)	
Gender				
Men *N* = 543	*N* = 114	*N* = 343	*N* = 86	
Prevalence, *N* (%)	11 (9.6)	30 (8.7)	4 (4.7)	
Nonadjusted	**3.62 (1.76–7.43)**	1.00	1.15 (0.40–3.27)	**0.002**
Multivariable-adjusted^§^	**3.79 (1.67–8.48)**	1.00	0.87 (0.29–2.61)	**0.005**
Women *N* = 758	*N* = 147	*N* = 436	*N* = 175	
Prevalence, *N* (%)	8 (5.4)	20 (4.6)	6 (3.4)	
Nonadjusted	**5.29 (2.16–13.0)**	1.00	1.26 (0.51–3.15)	≤**0.001**
Multivariable-adjusted^§^	**4.00 (1.43–11.2)**	1.00	1.19 (0.46–3.05)	**0.028**
Total *N* = 1,301	*N* = 261	*N* = 779	*N* = 261	
Prevalence, *N* (%)	19 (7.3)	50 (6.4)	10 (3.8)	
Nonadjusted	**4.45 (2.53–7.82)**	1.00	1.08 (0.54–2.13)	**<0.001**
Multivariable-adjusted^§^	**3.50 (1.87–6.55)**	1.00	0.94 (0.47–1.91)	**<0.001**

%dSUA, percentage change in serum uric acid; CI, confidence interval. Multivariable-adjusted for gender, age, body mass index, smoking habits, drinking habits, history of cardiovascular disease, hypertension, hypertriglyceridemia, low HDL-cholesterolemia, hyper LDL-cholesterolemia, diabetes, chronic kidney disease, hyperuricemia. Bolded numbers indicate significance (*p* < 0.05).

**Table 3:** Hazard ratios and 95% CIs for all-cause mortality according to %dSUA quintiles by subanalysis.

Baseline characteristics	*N*	%dSUA quintiles			*P* for trend
		Hazard ratio (95% CI)			
		Quintile 1	Quintiles 2–4	Quintile 5	
		<−1.88% (year)	−1.87 to 2.50% (year)	≥2.51% (year)	
Age					
<65 years	646	**5.82 (2.07–16.4)**	1.00	0.75 (0.17–3.38)	**0.003**
≥65 years	655	**2.77 (1.21–6.37)**	1.00	0.98 (0.43–2.22)	**0.043**
Body mass index					
<22.0 kg/m^2^	496	**7.59 (2.13–27.0)**	1.00	2.93 (1.05–8.18)	**0.005**
≥22.0 kg/m^2^	805	**3.08 (1.46–6.48)**	1.00	0.42 (0.13–1.42)	**0.002**
Medication^#^					
Absence	818	**4.32 (1.98–9.44)**	1.00	0.33 (0.08–1.39)	≤**0.001**
Presence	483	**2.85 (1.03–7.90)**	1.00	1.59 (0.62–4.04)	0.334
Chronic kidney disease					
eGFR <60 mL/min/1.73 m^2^	95	1.80 (0.22–14.8)	1.00	20.0 (1.09–369)	0.112
eGFR ≥60 mL/min/1.73 m^2^	1,206	**4.07 (2.06–8.06)**	1.00	0.73 (0.32–1.66)	**<0.001**
Hyperuricemia					
SUA <6.0 for men or <5.0 for women	806	**4.67 (1.94–11.2)**	1.00	0.91 (0.41–2.03)	≤**0.001**
SUA ≥6.0 for men or ≥5.0 for women	495	**3.39 (1.26–9.16)**	1.00	3.08 (0.62–15.2)	**0.042**

%dSUA, percentage change in serum uric acid; CI, confidence interval. ^#^Antihypertensive medication, antidyslipidemic medication, antidiabetic medication. Multivariable-adjusted for gender, age, body mass index, smoking habits, drinking habits, history of cardiovascular disease, hypertension, hypertriglyceridemia, low HDL-cholesterolemia, hyper LDL-cholesterolemia, diabetes, chronic kidney disease, and hyperuricemia. Bolded numbers indicate significance (*p* < 0.05).

## Data Availability

The datasets analyzed in this study can be available by the corresponding author (Ryuichi Kawamoto, rykawamo@m.ehime-u.ac.jp) upon reasonable request. The figure data and related data used to support the findings of this study are included within the article.
